# Characterization of the circulating transcriptome expression profile and identification of novel miRNA biomarkers in hypertrophic cardiomyopathy

**DOI:** 10.1186/s40001-023-01159-7

**Published:** 2023-06-30

**Authors:** Lanyan Guo, Yue Cai, Bo Wang, Fuyang Zhang, Hang Zhao, Liwen Liu, Ling Tao

**Affiliations:** 1 Department of Cardiology, Xijing Hospital, the Fourth Military Medical University, 127 Changle West Road, Xi’an, 710032 Shaan Xi China; 2 Department of Ultrasound, Xijing Hospital, the Fourth Military Medical University, 127 Changle West Road, Xi’an, 710032 Shaan Xi China

**Keywords:** Hypertrophic cardiomyopathy, miRNAs, Weighted correlation network analysis, Gene set enrichment analysis, Competing endogenous RNA network

## Abstract

**Background:**

Hypertrophic cardiomyopathy (HCM), one of the most common genetic cardiovascular diseases, but cannot be explained by single genetic factors. Circulating microRNAs (miRNAs) are stable and highly conserved. Inflammation and immune response participate in HCM pathophysiology, but whether the miRNA profile changes correspondingly in human peripheral blood mononuclear cells (PBMCs) with HCM is unclear. Herein, we aimed to investigate the circulating non-coding RNA (ncRNA) expression profile in PBMCs and identify potential miRNAs for HCM biomarkers.

**Methods:**

A Custom CeRNA Human Gene Expression Microarray was used to identify differentially expressed (DE) mRNAs, miRNAs, and ncRNAs (including circRNA and lncRNA) in HCM PBMCs. Weighted correlation network analysis (WGCNA) was used to identify HCM-related miRNA and mRNA modules. The mRNAs and miRNAs from the key modules were used to construct a co-expression network. Three separate machine learning algorithms (random forest, support vector machine, and logistic regression) were applied to identify potential biomarkers based on miRNAs from the HCM co-expression network. Gene Expression Omnibus (GEO) database (GSE188324) and experimental samples were used for further verification. Gene set enrichment analysis (GSEA) and competing endogenous RNA (ceRNA) network was used to determine the potential functions of the selected miRNAs in HCM.

**Results:**

We identified 1194 DE-mRNAs, 232 DE-miRNAs and 7696 DE-ncRNAs in HCM samples compared with normal controls from the microarray data sets. WGCNA identified key miRNA modules and mRNA modules evidently associated with HCM. We constructed a miRNA‒mRNA co-expression network based on these modules. A total of three hub miRNAs (miR-924, miR-98 and miR-1) were identified by random forest, and the areas under the receiver operator characteristic curves of miR-924, miR-98 and miR-1 were 0.829, 0.866, and 0.866, respectively.

**Conclusions:**

We elucidated the transcriptome expression profile in PBMCs and identified three hub miRNAs (miR-924, miR-98 and miR-1) as potential biomarkers for HCM detection.

**Supplementary Information:**

The online version contains supplementary material available at 10.1186/s40001-023-01159-7.

## Background

Hypertrophic cardiomyopathy (HCM), a genetic disease of the sarcomere, is widely distributed with a prevalence of 1:200–1:500 in the general population, but owned a high undetected rate [1, 2]. Owing to morphological and clinical heterogeneity, many patients have no evident symptoms throughout their lifetime, but 30–40% of HCM patients will experience at least one adverse cardiac event, including progressive heart failure (HF), thromboembolic events, or sudden cardiac death (SCD) due to ventricular tachycardia [1, 3]. With the advancement in the standardized management of HCM, the survival rate of HCM patients in dedicated HCM centers has become similar to that of the normal population [4, 5]. Therefore, prompt identification of the disease and dedicated referrals is important for maintaining such success.

A variety of mechanisms are involved in HCM. Inflammation occurs throughout HCM, and alleviation of inflammatory responses may reduce myocardial fibrosis [6, 7]. Monocyte activation mediates the aggregated infiltration and activation of monocytes–macrophages in the myocardium, which in turn promotes myocardial hypertrophy and remodeling [8]. Oxidative stress is also involved in HCM. Myocardial hypercontraction in HCM increases the energy demand, which in turn leads to increased production of reactive oxygen species as byproducts of oxidative phosphorylation [9]. Increased ROS and insufficient antioxidants place mitochondria in a state of oxidative stress, leading to mitochondrial damage [10]. Inflammation, oxidative stress, energy metabolism disorders and mitochondrial dysfunction are all involved in HCM.

Surprisingly, in the completed human genome, protein-coding transcripts account for less than 3% of the entire genome, which means that more than 97% of transcripts are noncoding RNAs [11]. miRNAs, a class of small noncoding RNAs, negatively regulate target mRNAs and regulate nearly 2/3 of known human genes [12]. Many studies have confirmed that miRNAs are of great significance in the pathogeneses of cardiovascular diseases; for example, they help regulate myocardial fibrosis, pathological myocardial hypertrophy, and HF [13–15]. In hypertrophic cardiomyocytes, a reciprocal repression mechanism between CHRF and miR-489 was first described by Wang: CHRF promotes myocardial hypertrophy as an endogenous sponge of miR-489 [16]. miRNAs are highly conserved, so they have potential to be used as biomarkers for physiological and pathological processes [17]. Extracellular circulating miRNAs are remarkably stable and protected from endogenous RNase activity, even under several extreme conditions, such as repeated freeze–thaw cycles, boiling, and long-term storage [18]. Furthermore, over 80% of genes expressed in heart muscle are also expressed in blood. Blood cells can act as sentinels for the diagnosis or prognosis of disease (the “Sentinel Principle”). Peripheral blood is an ideal surrogate tissue, because it is readily obtainable, provides a large biosensor pool in the form of gene transcripts and can be used to detect alterations in gene transcript levels in response to changes in the macro- and micro-environments [19].

Recent studies have identified potential competing endogenous RNA (ceRNA) regulatory networks in HCM myocardial tissue [20]. However, unfortunately, there are no circulating miRNAs that are reliable biomarkers for the detection of HCM, which limits their widespread application in clinical practice. Peripheral blood mononuclear cells (PBMCs) play an important role in the immune-inflammation response, so we aimed to determine the miRNA profile in PBMCs and explore potential miRNA biomarkers for HCM.

In this study, we analyzed circulating transcriptome expression profile in PBMCs with HCM and identified key miRNA and mRNA modules by weighted gene network co-expression analysis (WGNCA). Ultimately, a total of three key miRNAs with diagnostic potential were screened and further validated based on Gene Expression Omnibus (GEO) database and by RT-qPCR. Then, we predicted the potential ceRNA regulatory network of three key miRNAs.

## Materials and methods

### Sample collection

A total of 22 blood samples, including 6 from normal controls (NCs) and 16 from HCM patients (8 hypertrophic obstructive cardiomyopathy [HOCM] and 8 hypertrophic nonobstructive cardiomyopathy [HNCM]), were collected when the individuals visited the International Cooperation Center for Hypertrophic Cardiomyopathy, Xijing Hospital, between September 2019 and January 2020. Plasma was obtained after centrifugation, at 2000 rpm for 10 min, and stored in sterile tubes at − 80 °C until further processing. PBMCs were extracted with a human PBMC isolation kit (Solarbio). Total RNA extraction from PBMCs was performed by TRIzol LS Reagent (Invitrogen), following the manufacturer’s instructions. HCM was diagnosed according to the 2014 ESC guidelines on the diagnosis and management of HCM [21]. Which is defined as a maximum wall thickness (MWT) ≥ 13 mm or ≥ 15 mm in any ventricular segment for individuals with and without a family history of HCM, respectively, with the absence of any abnormal secondary causes capable of producing such a magnitude of hypertrophy, such as uncontrolled hypertension or aortic stenosis (AS), etc. The left ventricular outflow tract gradient (LVOTG) was measured with color-guided continuous-wave Doppler Echo at rest (Philips Medical Systems) and during provocation, such as during Valsalva maneuver or exercise stress with a supine bicycle (semi-recumbent and tilting bicycle Ergometer; Lode BV) [22, 23]. The presence of a peak left ventricular LOVTG ≥ 30 mmHg at rest or during exercise provocation was considered indicative of HOCM, while HNCM was defined by LVOTG < 30 mmHg at rest and exercise stress [21, 24]. NCs were individuals who were visited for screening HCM, but were eventually precluded from having HCM due to normal ventricular wall thickness and normal genotype. All participants underwent transthoracic two-dimensional and Doppler echocardiography independently conducted by two experienced ultrasound technicians. All procedures were conducted in accordance with the guidelines of the American Society of Echocardiography (ASE) [25]. Genetic testing was conducted and analyzed for every participant by next generation sequencing (Mygenostics). The test and analysis of ceRNA was completed with a custom ceRNA human gene expression microarray (2*400 k) by CapitalBio Technology.

This study was approved by the ethics committee of Xijing Hospital, Fourth Military Medical University, and complied with the Declaration of Helsinki. Written informed consent was obtained from all participants.

### Microarray data analysis

Differentially expressed genes (DEGs) in the microarray were screened using the R package limma. *P* < 0.05 and a fold change ≥ 1.5 were considered the cutoffs for DEGs.

### miRNA function prediction

miRNA-targeted mRNAs were predicted using miRWalk v2.0 (http://mirwalk.umm.uni-heidelberg.de) with potential binding positions in the 3’ UTR [26].

### Function enrichment analysis

Gene Ontology (GO) biological process (BP) and Kyoto Encyclopedia of Genes and Genomes (KEGG) pathway enrichment analyses were performed by the R package clusterProfiler with *P* < 0.05 [27]. The R package GOplot was used to show the results of GO BP [28]. Gene set enrichment analysis (GSEA) was performed using KEGG pathway annotation data and analyzed using the package clusterProfiler, and a ridgeline plot was used to show the results of GSEA [29].

### WGCNA

All miRNAs (2798 miRNAs) and DE-mRNAs from HCM and NC imported for weighted gene co-expression network analysis (WGCNA) were merged to construct the co-expression network. First, to construct the correlation network, the power of *β* = 6 for miRNA data and *β* = 9 for mRNA data realizes the scale-free topology criterion of R^2^ > 0.8 in this study (Additional file 1: Figure S1). Second, the average linkage hierarchical clustering method was applied to cluster DEGs into different modules with different colors. The cut height was set as 0.3, and the number that was selected as the minimum number of genes in each module. Different modules are shown with different colors, except the gray module, which contained DEGs that could not be merged. Third, the correlation between each module and HCM-related traits was calculated using Pearson correlation. The module with a *P* < 0.05 and the highest correlation coefficient was screened out for further analysis [30, 31].

### Screening for HCM potential biomarkers

First, the R package caret was used to divide HCM and NC samples into a training set and a test set. Then, three machine learning algorithm including random forest (RF), support vector machine (SVM) and logistic regression (LR) were used to build classifiers for HCM based on the expression of miRNAs from the miRNA‒mRNA co-expression network via the R packages randomForest, glmnet, and e1071, respectively. The R package pROC was used to display the receiver operating characteristic (ROC) curves and calculate the area under the curve (AUC). The best-performing classifier with the highest AUC was utilized to examine the test set and independent external data (GEO accession: GSE188324 data set) containing miRNA expression assays with 24 HCM patients and 11 NCs. In addition, the best optimal characteristic variables were used for further experimental verification.

### Real-time quantitative polymerase chain reaction (RT-qPCR)

The miRNAs contained in the PBMCs were extracted using the RNeasy Mini Kit for Small RNA (217004, Qiagen) followed with the standard procedures. The reverse transcription of pre-miRNAs and mature miRNAs was conducted using the PrimeScript™ RT Reagent Kit (RR047A, Takara) and miScript II RT Kit (218161, Qiagen). RT-qPCR was performed using the TB Green Premix ExTaq II Kit (RR 820A, Takara), and U6 was set as the expression control. RT-qPCR was conducted on a QuantStudio 5 (Thermo Fisher Scientific) with the following protocol: 95 °C for 5 min, 45 cycles of 95 °C for 10 s, and 60 °C for 30 s. The relative expression of miRNA expression was calculated using the comparative cycle threshold 2^−ΔΔCt^ method. The primer sequences were listed in the Additional file 1: Table S1.

### Functional prediction of clinical miRNAs for HCM

Gene set variation analysis (GSVA) was used to estimate the score of all selected miRNA expression levels for each HCM sample. Then, GSEA was used to perform functional prediction between the selected miRNAs with high expression levels and the selected miRNAs with low expression levels in HCM patients.

### Construction of the ceRNA network

According to the ceRNA hypothesis, miRNAs can induce gene silencing and downregulate gene expression by binding to mRNA, and lncRNAs can enrich miRNA binding sites and act as miRNA sponges, leading to changes in the expression levels of miRNA‒target mRNAs. Differentially expressed ncRNAs (DE-ncRNAs, fold change ≥ 1.5,* P* < 0.05) in HCM with a highly positive correlation coefficient (correlation coefficient > 0.9) for mRNAs from mRNA modules and targeted by miRNAs based on miRWalk that could bind the same miRNAs with related mRNAs based on RNA hybrid prediction were used to construct ceRNA networks. Consequently, the result was used to construct a ceRNA network in Cytoscape.

### Statistical analysis

Continuous variables are presented as the mean (standard deviation, SD) or median (interquartile range, IQR) when appropriate, and categorical variables are presented as counts (percentages). The independent *t* test was used for the comparison of two continuous variables with a normal distribution, and nonnormally distributed data were compared using a two-sided nonparametric Mann–Whitney *U* test.* χ*^2^ or Fisher’s exact test was used for the comparison of unordered categorical variables. ROC were performed and the area under the ROC (AUC) with 95% confidence interval, sensitivity and specificity were calculated to evaluate the key miRNAs as potential HCM biomarkers.

All data were analyzed by SPSS (version 26.0) and R software (version 4.1.1). A two-tailed* P* < 0.05 was considered to indicate statistical significance.

## Results

### Baseline characteristics

The baseline clinical characteristics of a total of 22 samples, including 6 NC and 16 HCM (8 HOCM and 8 HNCM) were presented in Table 1. In the two HCM subgroups, 4 for sarcomere gene positive, including 2 for MYH7 and 2 for MYBPC3 positive, respectively; and the 4 others were gene negative. There was no difference in mean age, sex, and blood pressure between HCM and NC, HOCM and HNCM (all *P* > 0.05). The mean MWT was 24.69 mm, and LVOTGmax was 49.5 mm Hg in HCM patients.

**Table 1:** Baseline characteristics

All (*N* = 22)				HCM (*N* = 16)			HOCM (*N* = 8)			HNCM (*N* = 8)		
Parameters	HCM *N* = 16	NC *N* = 6	*P*	HOCM *N* = 8	HNCM *N* = 8	*P*	Gene ( +) *N* = 4	Gene (−) *N* = 4	*P*	Gene ( +) *N* = 4	Gene (−) *N* = 4	*P*
Age, years	43.19 (10.18)	35.67 (12.99)	0.167	43.50 (8.72)	42.88 (12.08)	0.907	42.50 (7.33)	44.50 (11.00)	0.772	38.25 (10.40)	47.50 (13.23)	0.314
Sex (N, %)	13 (81.25)	4 (66.67)	0.585	6 (75.00)	7 (87.50)	> 0.999	3 (0.75)	4 (1.00)	> 0.999	2 (0.50)	4 (1.00)	0.429
BMI, kg/m^2^	25.89 (2.71)	22.80 (2.06)	0.020	25.19 (2.53)	26.59 (2.86)	0.318	24.02 (2.74)	26.36 (1.95)	0.213	24.61 (1.65)	28.57 (2.43)	0.035
SBP, mmHg	123.69 (15.91)	127.17 (10.21)	0.625	116.38 (17.14)	131 (11.25)	0.063	107.75 (16.92)	125.00 (14.17)	0.169	128.25 (16.26)	133.75 (3.30)	0.532
DBP, mmHg	73.88 (13.10)	78.50 (6.75)	0.424	68 (16.33)	79.75 (4.74)	0.071	63.25 (5.85)	72.75 (22.97)	0.453	78.25 (5.56)	81.25 (3.95)	0.413
MWT, mm	24.69 (5.69)	8.17 (0.98)	< 0.001	26.00 (5.81)	23.38 (5.63)	0.374	25.50 (7.77)	26.50 (4.20)	0.828	24.75 (5.50)	22.00 (6.22)	0.532
LVOTG_rest_, mmHg	21.00 (4.75, 72.00)	3.00 (2.75, 3.25)	0.001	24.00 (22.00, 30.75)	5.50 (4.00, 12.00)	0.001	33.50 (28.75, 66.00)	86.50 (72.00, 102.50)	0.081	5.50 (4.00, 12.25)	6.50 (3.25, 12.00)	0.767
LVOTG_max_, mmHg	49.50 (13.48, 120.75)	–		119.50 (96.75, 126.78)	13.95 (8.15, 19.25)	0.001	108.00 (78.00, 120.75)	125.85 (103.00, 135.43)	0.149	13.95 (9.25, 18.73)	12.80 (8.15, 25.25)	0.885
LAD, mm	41.88 (6.33)	33.33 (1.03)	< 0.001	43.75 (7.11)	40.00 (5.24)	0.249	48.00 (5.35)	39.50 (6.40)	0.088	39.00 (4.97)	41.00 (6.06)	0.628
RAD, mm	32.31 (4.44)	30.83 (0.98)	0.226	32.50 (4.78)	32.13 (4.39)	0.873	33.75 (4.35)	31.25 (5.50)	0.503	31.25 (5.19)	33.00 (4.00)	0.612
EDV, ml	78.81 (15.01)	71.50 (2.95)	0.080	78.13 (10.59)	79.50 (19.22)	0.862	77.75 (12.42)	78.50 (10.34)	0.929	75.00 (26.99)	84.00 (8.91)	0.550
ESV, ml	31.31 (9.66)	29.33 (1.86)	0.445	31.00 (6.91)	31.63 (12.33)	0.902	30.00 (5.23)	32.00 (9.02)	0.714	29.50 (15.84)	33.75 (9.58)	0.662
E/A	0.85 (0.29)	1.19 (0.20)	0.019	0.81 (0.27)	0.89 (0.33)	0.623	0.93 (0.30)	0.70 (0.20)	0.256	0.73 (0.14)	1.05 (0.41)	0.220
E/e’	12.75 (10.55, 15.50)	6.90 (6.30, 7.29)	0.002	15.71 (6.87)	11.88 (2.28)	0.157	14.08 (3.48)	17.34 (9.54)	0.544	11.45 (3.10)	12.30 (1.44)	0.637
LVEF (%)	60.81 (5.37)	58.67 (1.97)	0.185	60.63 (4.75)	61.00 (6.26)	0.895	61.50 (2.89)	59.75 (6.50)	0.640	62.00 (6.33)	60.00 (6.98)	0.686

Data are expressed as mean (SD), otherwise specified

*HOCM* obstructive hypertrophic cardiomyopathy, *HNCM* non-obstructive hypertrophic cardiomyopathy, *BMI* body mass index; *SBP* systolic blood pressure, *DBP* diastolic blood pressure, *MWT* maximum wall thickness, *LVOTGrest* the maximum left ventricular outflow tract gradient (at rest), *LVOTGmax* the maximum LVOTG at provocation, *LAD* transverse diameter of left atrium, *RAD* transverse diameter of right atrium, *EDV* end-diastolic volume, *ESV* end-systolic volume, *E* peak flow velocity of early diastolic wave (mitral valve), *A* peak flow velocity of late diastolic wave (mitral valve), *E’* peak flow velocity of early diastolic wave (mitral annular), *LVEF* left ventricular ejection fraction

### Identification of DEGs

**Fig. 1:**
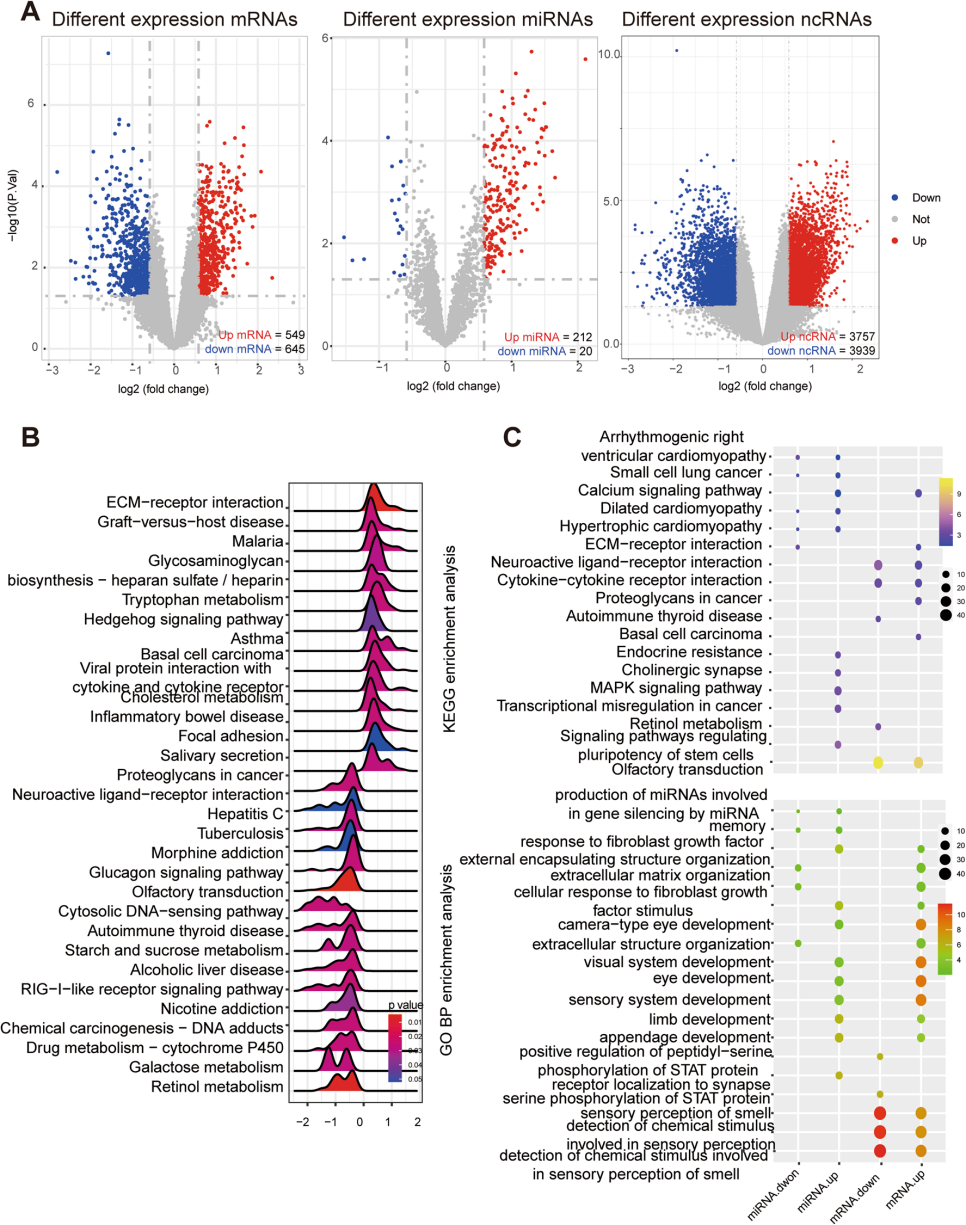
Transcriptome profile analyses of HCM. **A** Volcano plots of DE-mRNAs, DE-miRNAs and DE-ncRNAs (including DE-lncRNAs and DE-circRNAs) between the HCM and NC groups. **B** Ridge plots of the GSEA results based on KEGG between the HCM and NC groups. *X*-axis showed the log2 (fold change) of per genes present in each KEGG terms, with positive values indicating up-regulated expression and negative values as down-regulated expression in HCM. Peaks are colored based on *P* values as shown by the legend. **C** Enrichment analysis of DE-mRNAs and DE-miRNAs based on KEGG (top) and GO BP (bottom) terms. The dot size showed the number of genes which enriched in the KEGG or GO BP terms, and the color represents the *P* values of the KEGG or GO BP terms

To explore the profiles of differentially expressed (DE) mRNAs, miRNAs, and ncRNAs between HCM and NC, a total of 1194 mRNAs (549 upregulated and 645 downregulated), 232 miRNAs (212 upregulated and 20 downregulated) and 7696 ncRNAs **(**3757 upregulated and 3939 downregulated) were identified (Fig. 1A and Additional file 2: Table S2). Compared with NCs, HCM patients exhibited an inflammatory state, with overall upregulation of pathways, such as ECM − receptor interaction, cytokine − cytokine receptor interaction and inflammatory bowel disease, based on GSEA. In addition, metabolic disorders were also evident in HCM, such as tryptophan metabolism upregulation and starch and sucrose metabolism downregulation. Furthermore, we investigated the potential biological functions of DE-miRNAs and DE-mRNAs through KEGG and GO enrichment analyses. Unlike DE-mRNAs, DE-miRNAs were rarely associated with signaling molecules and interactions, such as cytokine − cytokine receptor interactions and neuroactive ligand − receptor interactions. DE-miRNAs and DE-mRNAs were both involved in cell development and the calcium signaling pathway. Moreover, DE-miRNAs were directly involved in HCM pathogenesis-related pathways, such as HCM and dilated cardiomyopathy (Fig. 1B, C). These results suggest that transcriptome alterations, especially those of miRNAs in PBMCs, may have an important impact on the pathogenesis of HCM.

### Subgroup analysis in HCM

**Fig. 2:**
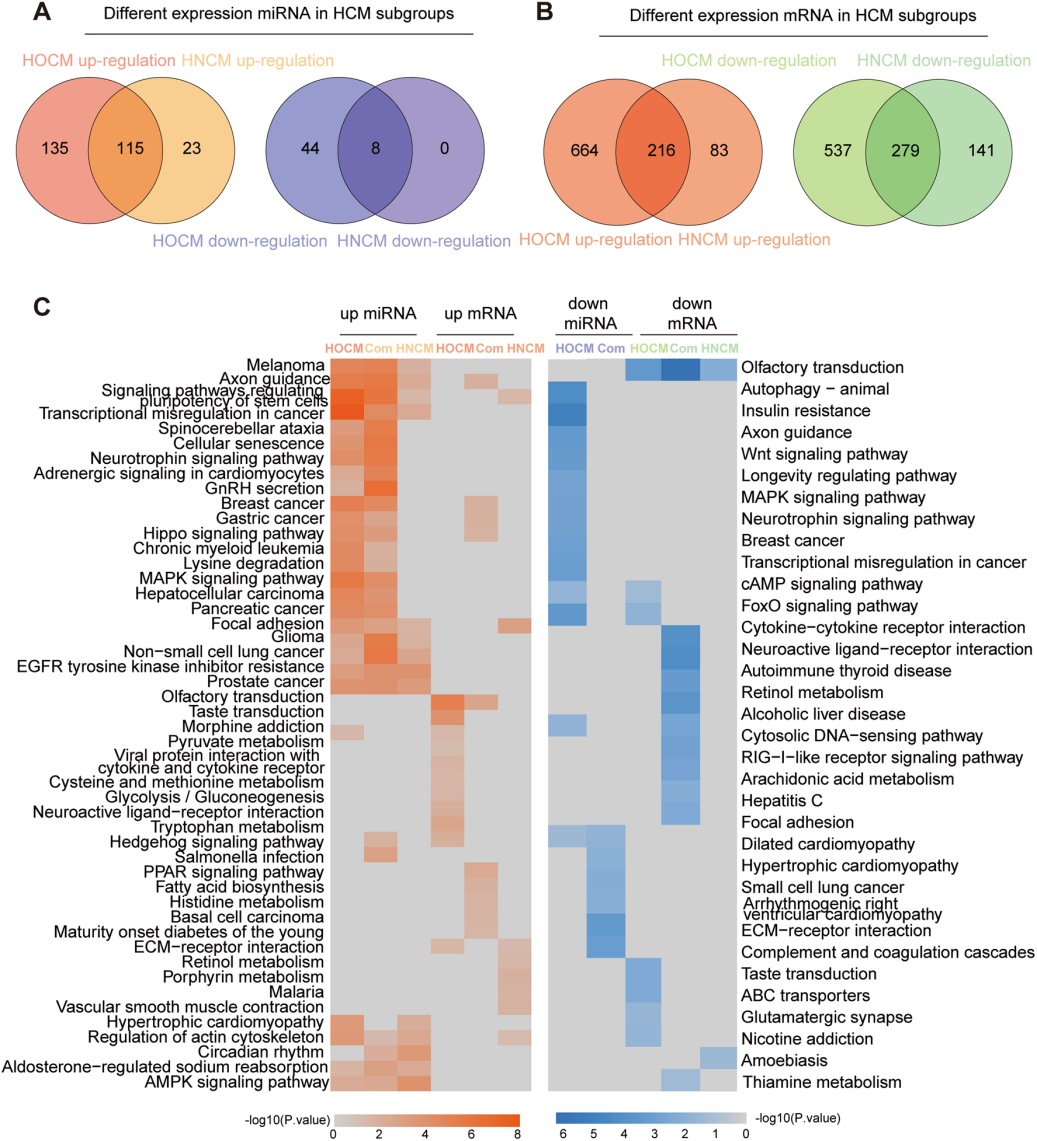
KEGG pathways in different HCM subgroups. Venn plot showing the common and unique DE-miRNAs (**A**) and DE-mRNAs (**B**) in different HCM subgroups. **C** Heatmap showing the *P* values of the top 5 significant upregulated (left) and downregulated (right) KEGG pathways based on the common and unique DE-miRNAs and DE-mRNAs in different HCM subgroups, respectively. The cell color represents the value of − log10(*P* values) in each KEGG term. *HOCM* obstructive hypertrophic cardiomyopathy; *HNCM* non-obstructive hypertrophic cardiomyopathy; Com, common NCs

To further explore the transcriptome in the HCM subtypes, we determined the DE-mRNAs and DE-miRNAs in each subgroup (Fig. 2A, B). The results showed that compared with HNCM patients, HOCM patients exhibited more unique DE-mRNAs and DE-miRNAs, especially downregulated DE-miRNAs. Further analysis showed that upregulated miRNAs in both HNCM and HOCM had similar biological functions, while upregulated mRNAs in HOCM were more relevant to inflammatory pathways than those in HNCM. Distinct metabolic changes were also observed between HOCM and HNCM. In HOCM, downregulated DE-mRNAs exhibited abnormal suppression of the AMP signaling pathway and FOX signaling pathway, while downregulated DE-miRNAs were mainly involved in cell proliferation and apoptosis-related pathways (Fig. 2C).

### Identification of HCM-related key miRNA modules and mRNA modules via WGCNA

**Fig. 3:**
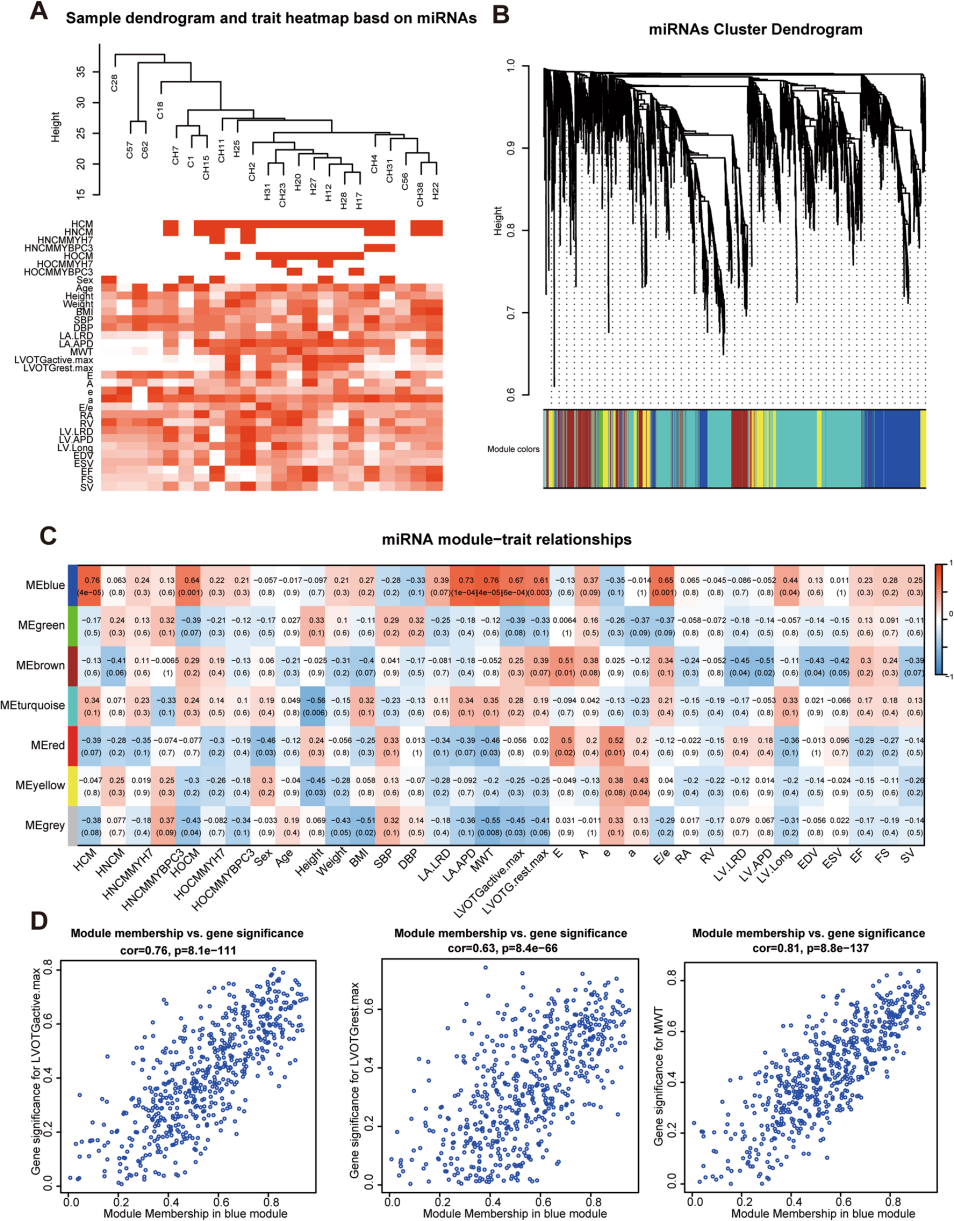
Identified HCM-related miRNA modules based on WGCNA. **A** Cluster dendrogram of samples based on the expression of all miRNAs. **B** Cluster dendrogram of co-expression modules by hierarchical clustering of miRNAs. Different colors represent different modules which contains a group of highly associated genes. **C** Heatmap of the correlation between miRNA modules and HCM-related clinical features. The cell is colored by correlation coefficient according to the color legend. **D** Correlation of miRNAs in blue miRNA module with LVOTGmax and MWT, all* P* < 0.05. HCM, hypertrophic cardiomyopathy. LVOTGrest.max, the maximum left ventricular outflow tract gradient at rest. LVOTGactive.max, the maximum left ventricular outflow tract gradient after provocation. *MWT* maximum wall thickness

To identify the hub miRNAs and hub mRNAs in HCM, WGCNA was used to determine key miRNA modules and mRNA modules. First, all miRNAs (a total of 2798 miRNAs) were used to construct the co-expression network. The clustering results showed that all miRNAs were still able to distinguish NCs from patients with HCM (Fig. 3A). After WGCNA, these miRNAs were clustered into a total of 7 modules using the average linkage hierarchical clustering algorithm (Fig. 3B and Additional file 2: Table S3). Subsequently, we detected the correlation between these miRNA modules and clinical traits. All the related clinical traits, including the maximum LVOTG (LVOTGmax) and maximum wall thickness (MWT), were positively correlated with the blue miRNA module (all* P* < 0.05), which indicated that the key miRNAs in the blue miRNA module were also important for the module itself and clinical characterization (Fig. 3C, D).

**Fig. 4:**
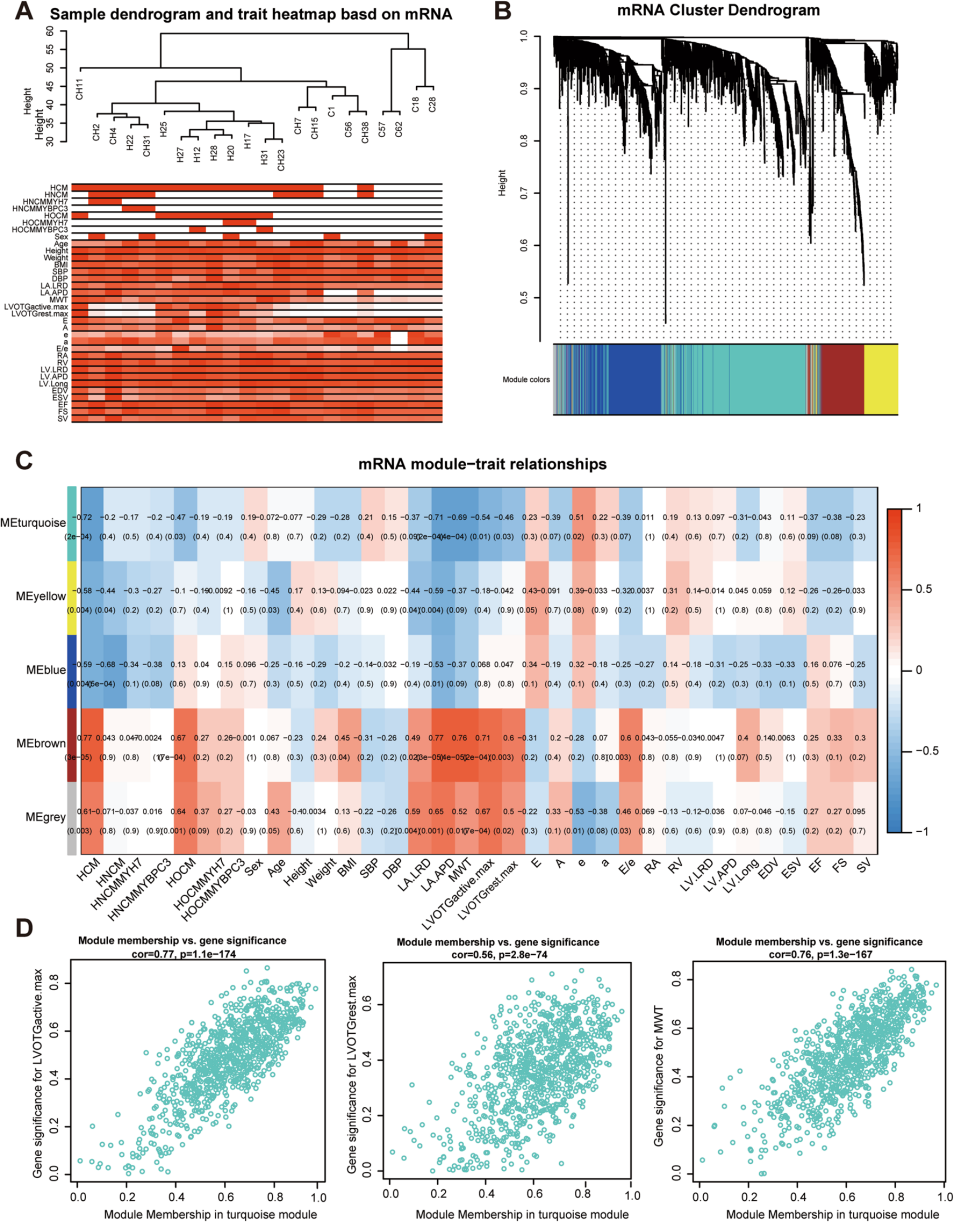
Identified HCM-related mRNA modules based on WGCNA. **A** Cluster dendrogram of samples based on DE-mRNA expression. **B** Cluster dendrogram of co-expression modules by hierarchical clustering of DE-mRNAs. Different colors represent different modules which contains a group of highly connected genes. **C** Heatmap of the correlation between mRNA modules and HCM-related clinical features. The cell is colored by correlation coefficient according to the color legend. **D** Correlation of mRNAs in turquoise mRNA modules with LVOTGmax and MWT, all *P* < 0.05

Then, we adopted the same method to identify key mRNA modules in HCM based on a total of 1992 DE-mRNAs. The clustering results showed that such DE-mRNAs were able to distinguish HCM from NC cases (Fig. 4A and Additional file 2: Table S4). After WGCNA, these mRNAs were clustered into a total of 5 modules which involved in different pathways **(**Fig. 4B and Additional file 1: Figure S2). Similarly, the genes in the turquoise mRNA module and clinical traits, including LVOTG and MWT, were positively correlated with great significance (all* P* < 0.05), so the turquoise mRNA module was identified as the key mRNA module (Fig. 4C, D).

### Construction of the key miRNA‒mRNA co-expression network in HCM

**Fig. 5:**
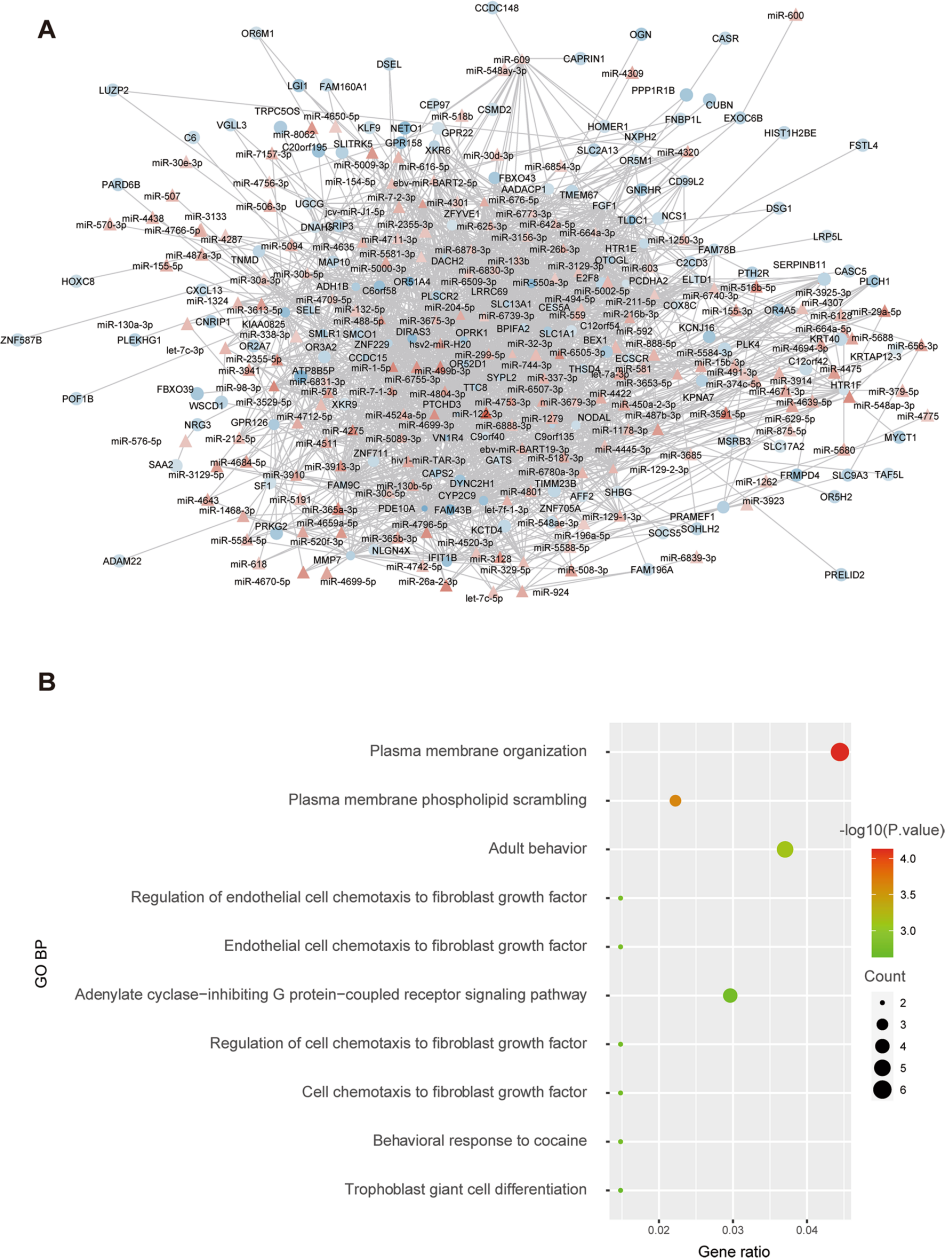
Identification of the miRNA‒mRNA co-expression network based on key miRNA modules and mRNA modules. **A** miRNA‒mRNA co-expression network based on key miRNA modules and mRNA modules. Circle represents mRNAs from key mRNA modules and triangle represents miRNAs from key miRNA modules. Red represents mRNAs or miRNAs up-regulation in HCM, and blue represents mRNAs or miRNAs down-regulation in HCM. **B** Results of GO BP enrichment analysis based on mRNAs from miRNA-mRNA co-expression network. The dot size indicated the number of genes which enriched in the GO BP terms, and the color represents the *P* values of the GO BP terms

Then, we constructed a co-expression network based on miRNAs and mRNAs from key modules to explore the potential relationship between the two key modules. Usually, miRNAs suppress the expression of mRNAs, so we selected the co-expression pairs with a negative correlation between miRNAs and mRNAs to construct a co-expression network including 185 miRNAs and 355 mRNAs in Cytoscape (Fig. 5A). Further analysis showed that the mRNAs from these networks were involved in regulating G protein − coupled receptor-related signaling pathway and fibroblast growth factor-related biological process such as cell chemotaxis to fibroblast growth factor **(**Fig. 5B**)**.

### Screening and validation of potential biomarkers from key miRNAs

**Fig. 6:**
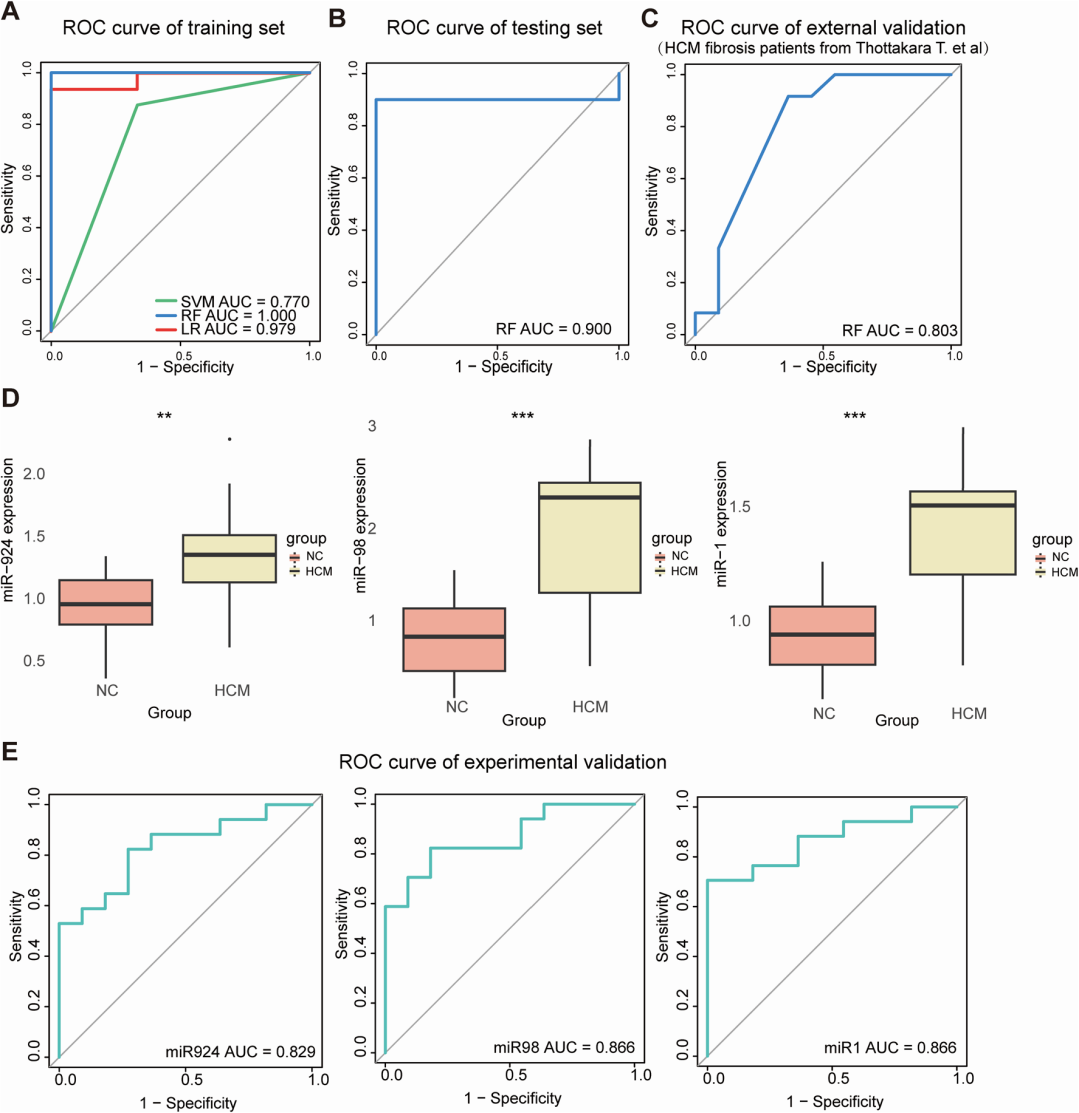
Screening and validation of potential biomarkers for the detection of HCM from key miRNAs. **A** ROC curves of the training set based on three ML (SVM, RF, LR) algorithms for distinguishing HCM patients from NCs. **B** ROC curves of the testing set based on the RF module. **C** ROC curves of external validation (GSE188324) based on the RF module. **D** Boxplot showing the expression levels of three miRNAs which were important variables based on RF in HCM patients and NCs. ^**^ and ^***^ indicated *P* values < 0.01 and < 0.001. **E** ROC curves of experimental validation based on the RT-qPCR results of three miRNAs for distinguishing HCM patients from NCs

To screen potential biomarkers for HCM, we randomly divided these miRNAs into training and testing groups, used three different machine learning (ML) algorithms (including SVM, RF and LR), and showed that all these miRNAs were able to discriminate HCM from NC by all 3 ML algorithms (AUC was 0.770 with SVM, 1.000 with RF, and 0.979 with LR, all AUC > 0.75) (Fig. 6A). The RF module has the highest AUC value compared with two others, and then the validation results of the test group and external validation (GSE188324) with RF module also showed the potential for the detection of HCM (AUC was 0.900 and 0.803, respectively, all AUC > 0.75) (Fig. 6B, C). Then, we screened 3 important variables based on RF and performed the next experimental validation, which showed that miR-924, miR-98 and miR-1 were all significantly elevated in HCM, which was consistent with our gene chip results (Fig. 6D and Additional file 1: Figure S3). The AUC of miR-924 was 0.829, that of miR-98 was 0.866, and that of miR-1 was 0.866, respectively (Fig. 6E).

### GSEA and ceRNA network construction for the three selected miRNAs

**Fig. 7:**
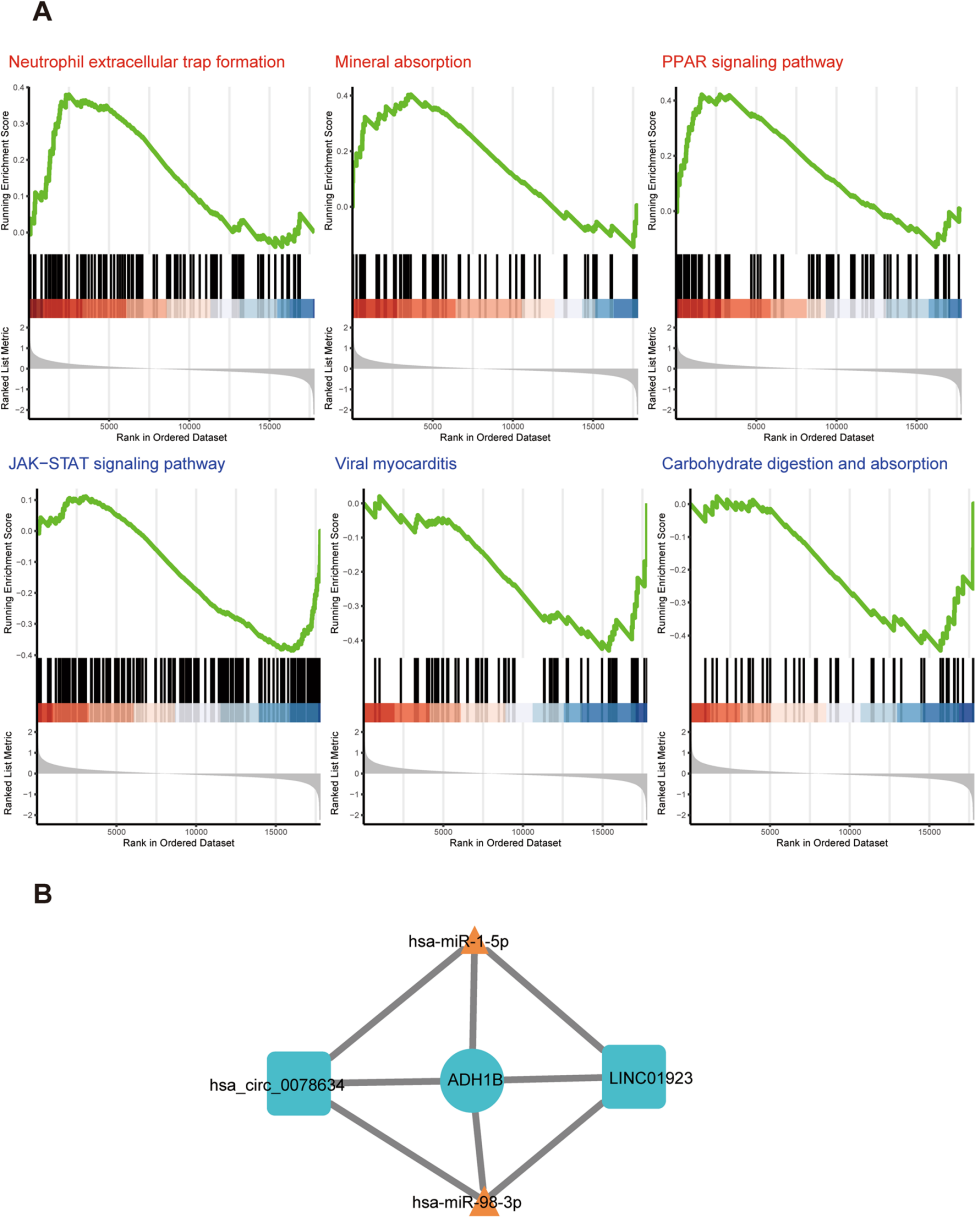
GSEA and ceRNA network of the three target miRNAs. **A** Upregulation pathway of three miRNAs in the high-expression group compared with the low-expression group (top). Downregulated pathways of the three miRNAs in the high-expression group compared with the low-expression group (bottom). **B** ceRNA network of miR-1-5p and miR-98-3p in HCM PBMCs

To explore the potential biological function and regulatory network of these three selected miRNAs with diagnostic performance, we assigned a specific score value for each HCM patient according to the overall expression levels of these three miRNAs. Then, HCM patients were divided into a high expression group and a low expression group. Through GSEA, it was found that in patients with high expression, genes related to mineral absorption and PPAR were upregulated, while genes related to the JAK–STAT pathway, sugar absorption and metabolism, and viral cardiomyopathy were downregulated (Fig. 7A). Furthermore, we constructed a potential ceRNA regulatory network of miRNAs on target mRNAs and found that miR-1 and miR-98 can regulate the expression of ADH1B through the ceRNA network. The lncRNA (LINC01923) and the circRNA (hsa-circ-0078634) could prevent miRNA binding with ADH1B (Fig. 7B).

## Discussion

In this study, we created a differentially expressed transcript atlas for PBMCs in HCM. Compared with NCs, HCM patients showed more abnormalities in inflammatory signaling, metabolic pathways and calcium pathways. Previous studies have shown that abnormalities of such pathways in cardiac muscle tissue play an important role in the development of HCM, presenting consistent changes in PBMCs and myocardial tissue in HCM [32]. Furthermore, DE-miRNAs in PBMCs are more relevant to HCM-related pathogenic pathways than DE-mRNAs, which suggests the importance of circulating miRNA alterations for the pathogenesis of HCM and their potential as diagnostic biomarkers and therapeutic targets.

It has been reported that HOCM accounts for 60–70% of all HCM cases [33]. Because of the distinct treatment approaches and prognoses, the discrimination of the two subgroups is also significant. Based on the transcriptome atlas, we found more significantly differentially expressed mRNAs in HOCM than in HNCM, and these DE-mRNAs were involved in metabolic, apoptosis-related and proliferation-related pathways. There were fewer DE-miRNAs between the two subtypes. These results indicated that there were different transcription factors and pathways between HOCM and HNCM, even though the upregulated miRNAs were functionally consistent between the two subgroups**.** This may suggest that the transcriptome of PBMCs is a potential biomarker to distinguish HOCM from HNCM, and may be useful to explore the different mechanisms between HOCM and HNCM.

WGCNA is an important bioinformatics analysis method that builds a network according to systematic gene expression to obtain genetic clusters that are functionally similar. We identified 7 miRNA modules and 5 mRNA modules based on WGCNA, analyzed the potential biological functions and correlation with clinical features of each module. The mRNAs in the turquoise mRNA module and miRNAs in the blue miRNA module were positively correlated with MWT and LVOTGmax. Based on key miRNA modules and mRNA modules correlated with clinical parameters, we identified a potential hub miRNA‒mRNA regulatory network and found that key miRNAs may regulate genes related to the JAK–STAT pathway, which is the key pathway in the pathogenesis of HCM [34].

In the present study, we identified three potential biomarkers based on ML and experimental verification, including miR-1, miR-98 and miRNA-924. miR-1 is a highly conserved miRNA with high expression in muscle tissue, particularly heart muscle. As a key regulator of cardiac hypertrophy, miR-1 may lead to a marked reduction in myocardial fibrosis, an improvement in calcium handling, inhibition of apoptosis, and inactivation of the mitogen-activated protein kinase signaling pathways [35]. Such results indicates that miR-1 has beneficial effects in preventing maladaptive ventricular remodeling, reversing pressure overload-induced cardiac hypertrophy and attenuating pathological remodeling. miR-1 is substantially downregulated in the rat hypertrophic left ventricle and in cardiomyocytes with phenylephrine-induced hypertrophy, and overexpression of miR-1 in hypertrophic cardiomyocytes reduces the cell size and attenuates the expression of hypertrophic markers, whereas silencing of miR-1 in cardiomyocytes results in the hypertrophic phenotype [36]. A recent study [37] has shown that oxidized miR-1 [7o(8)G-miR-1] can induce cardiac hypertrophy, indicating that position-specific oxidation of miR-1 could serve as a posttranscriptional mechanism to coordinate pathophysiological redox-mediated gene expression. It has been reported that some features in the plasma and cardiac tissues exhibit opposite alteration trends in HCM [38]. This may be explained by the phenomenon that cardiomyocyte death releases tissue-enriched miRNAs into the circulatory bloodstream, while in HCM, cardiomyocytes die in a staggered manner, in contrast to the abrupt and massive death observed in ischemic cardiac disease [39]. Similarly, we observed that circulating miR-1 was upregulated, consistent with the findings of others [40, 41], although was in contrast to the expression of hypertrophic cardiomyocytes [42]. Furthermore, its AUC was 0.866 in external samples (> 0.75, which indicates a clearly useful discrimination ability [43]), so the single miR-1 in PBMCs had a clearly useful ability to discriminate patients with HCM from NCs.

Cardiomyocyte hypertrophy, interstitial fibrosis, microvascular dysfunction and abnormal immune inflammatory response are pathological features of HCM [21, 44]. In this study, miR-98 was also evidently upregulated in HCM PBMCs. Upregulation of miR-98 dramatically ameliorates TGF-β1-induced collagen accumulation in cardiac fibroblasts, reflecting a protective effect of miR-98 overexpression against TGF-β1-induced cardiac fibrosis [45]. It has demonstrated that miR-98 negatively regulates cardiac hypertrophy. Since miR-98 is also upregulated by pressure overload in the mouse heart, it may act as a negative feedback regulator of Ang II-induced cardiac hypertrophy as well as other forms of cardiac hypertrophy [46]. Furthermore, expression of miR-98 is also increased by hypoxia. Zhang et al. has reported that hypoxia-induced bone marrow mesenchymal stem cells derived exosome miR-98-5p to protect against myocardial ischemia–reperfusion injury [47]. In addition, miR-98 protects endothelial cells against hypoxia/reoxygenation-induced apoptosis by targeting caspase-3 and attenuates cardiac ischemia/reperfusion (I/R) injury [48, 49]. Similarly, cardiac fibrosis, the activation of renin–angiotensin–aldosterone system (RAAS) and myocardial ischemia/hypoxia may partly explain the upregulation of miR-98 in HCM. It has also reported that miRNAs are associated with the immune inflammatory response. miR-98 is reported to suppress IL-10 expression in B cells of the heart, which plays an important role in myocarditis [50]. In brief, we found miR-98 in PBMCs was upregulated, which to some degree may reflect disease severity, and was associated with cardiomyocyte hypertrophy, cardiac fibrosis, microvascular dysfunction, cardiac ischemia, and immune inflammatory state. The AUC of miR-98 in PBMCs was 0.866 with experimental samples, so miR-98 has also a clearly useful ability to differentiate patients with HCM from NCs.

Previous research has shown that miR-924 inhibited cell proliferation as a tumor suppressor in non-small cell lung cancer (NSCLC) and hepatocellular carcinoma (HCC) [51, 52]. However, the role of miR-924 in heart disease is still unknown. In this study, the AUC of miR-924 in PBMCs was 0.829, which was also higher than 0.75. This indicated that miR-924 may also have a clearly useful capacity for identifying HCM. However, because of the lack of evidence about miR-924 in heart disease, more study is needed to verify its exact role in HCM.

Alcohol dehydrogenase-1B (ADH1B), as alcohol metabolizing genes, was differentially downregulated in many types of cancers, which exists glucose metabolism (Warburg Effect) [53]. Similarly, HCM myocardium is characterized as disordered energy metabolism, including impaired fatty acid oxidation and decreased glucose metabolism [9]. It has been reported that ADH1B ∗ 2 reduces the risk of NASH and fibrosis in adults with NAFLD regardless of alcohol consumption status [54]. Among East Asians, frequent alcohol use and individuals carrying ADH1B (A/A) polymorphisms was associated with worse global longitudinal strain, systolic and early diastolic strain rates [55]. It suggested that even moderate alcohol consumption imposed subclinical adverse effects on cardiac systolic/diastolic functions and cardiac remodeling, which was most evident in subjects carrying common alcohol metabolizing genes, including ADH1B. Based on such results, ADH1B was involved in energy metabolism, organ fibrosis, cardiac remodeling and function, which were all also the pathophysiological characteristics of HCM, so we speculate ADH1B may correlate with HCM. Nevertheless, the exact effect of ADH1B in HCM should be further conducted in the future study.

In this manuscript, we found ADH1B was to be regulated by miR-1 and miR-98 in peripheral blood, while the gene is mainly related to glucose absorption and energy metabolism. Therefore, we speculate that miR-1 and miR-98 may regulate energy metabolism by modulating the expression of ADH1B, and affect the pathogenesis of HCM. Furthermore, lncRNA (LINC01923) and circRNA (circ-0078634) can antagonize miR-1 and miR-98, which providing new perspectives for potential treatment in the future.

In the present study, we depicted the entire transcriptome profile in PBMCs with HCM and identified HCM-related key miRNA modules and mRNA modules via WGCNA. Then, 3 potential biomarker miRNAs (miR-1, miR-98, miR-924) were screened from the key miRNAs by different ML algorithms. Finally, further verification was performed with other external samples by ROC curve analysis. These 3 key miRNAs in PBMCs all had a potential discriminative ability for HCM.

## Conclusion

This study represents a preliminary attempt to delineate the entire transcriptome profile in PBMCs with HCM. Utilizing bioinformatics analysis and multiple ML algorithms, key miRNA modules and mRNA modules were identified, and 3 miRNAs, including miR-1, miR-98 and miR-924, were selected and further verified with external samples. Such key miRNAs in PBMCs have the clearly useful ability to be used as biomarkers for HCM detection.

## Limitations

This study had several limitations. First, the study was performed in a single center. Even though the center is one of the largest dedicated HCM centers in China, the sample size was relatively small. Furthermore, because circulating features are a reflection of the general disease state, whether the identified DE-miRNAs are involved in HCM pathophysiology and disease progression is still undetermined. Finally, although initial external validation by RT-qPCR was performed in this study and although all 3 key DE-miRNAs exhibited a satisfactory discrimination ability, research on a large prospective cohort is required to address the value for future clinical application in HCM detection and diagnosis.

## Supplementary Information

**Additional file 1: Figure S1**. Effects of different power values on the scale independence degree and mean connectivity of co-expression modules. A, Analysis of the scale-free fit index for various soft-threshold powers in miRNA expression matrix; the red line was set at 0.80. B, Analysis of mean connectivity for various soft-threshold powers in miRNA expression matrix；C, Analysis of the scale-free fit index for various soft-threshold powers in mRNA expression matrix; the red line was set at 0.80. D, Analysis of mean connectivity for various soft-threshold powers in mRNA expression matrix. **Figure S2**. Functional KEGG enrichment analysis of HCM-related mRNA modules. **Figure S3**. Top 5 characteristic variable filtering based on RF. A. Top 5 mean decrease accuracy miRNAs based on RF. B. Top 5 mean decrease Gini miRNAs based on RF. **Table S1**. Primer sequences for miRNAs.

**Additional file 2: Table S2**. DEGs in HCM compared with NC groups. **Table S3**. miRNAs in miR-modules. **Table S4**. mRNAs in mRNA-modules.

## Data Availability

All data generated or analyzed in this study are included in this published article. All the data are available from the corresponding author on reasonable request.

## Abbreviations

AUC: The area under the ROC curve
ceRNA: Competing endogenous RNA
DEG: Differentially expressed genes
DE-miRNAs: Differentially expressed miRNAs
DE-mRNAs: Differentially expressed mRNAs
GEO: Gene expression omnibus
GO: Gene Ontology
GSEA: Gene set enrichment analysis
HCM: Hypertrophic cardiomyopathy
HNCM: Non-obstructive hypertrophic cardiomyopathy
HOCM: Obstructive hypertrophic cardiomyopathy
KEGG: Kyoto Encyclopedia of Genes and Genomes
LR: Logistic regression
LVOTG: Left ventricular outflow tract gradient
ML: Machine learning
MWT: Maximum wall thickness
ncRNA: Non-coding RNA
PBMC: Peripheral blood mononuclear cells
RF: Random forest
ROC: Receiver operating characteristic
SVM: Support vector machine
WGCNA: Weighted gene network co-expression analysis
