# Supplementary material for: Characterization of the circulating transcriptome expression profile and identification of novel miRNA biomarkers in hypertrophic cardiomyopathy
Source: Eur J Med Res. 2023 Jun 30;28:205. doi: 10.1186/s40001-023-01159-7 (PMC10314611; doi:10.1186/s40001-023-01159-7)
Supplement: Supplementary file 1 — Additional file 1: Figure S1. Effects of different power values on the scale independence degree and mean connectivity of co-expression modules. A, Analysis of the scale-free fit index for various soft-threshold powers in miRNA expression matrix; the red line was set at 0.80. B, Analysis of mean connectivity for various soft-threshold powers in miRNA expression matrix；C, Analysis of the scale-free fit index for various soft-threshold powers in mRNA expression matrix; the red line was set at 0.80. D, Analysis of mean connectivity for various soft-threshold powers in mRNA expression matrix. Figure S2. Functional KEGG enrichment analysis of HCM-related mRNA modules. Figure S3. Top 5 characteristic variable filtering based on RF. A. Top 5 mean decrease accuracy miRNAs based on RF. B. Top 5 mean decrease Gini miRNAs based on RF. Table S1. Primer sequences for miRNAs. [file 40001_2023_1159_MOESM1_ESM.pdf]

Additional file figures and figure legends

Figure S1. Effects of different power values on the scale independence degree and mean connectivity of co-expression modules

A, Analysis of the scale-free fit index for various soft-threshold powers in miRNA expression matrix; the red line was set at 0.80. B, Analysis of mean connectivity for various soft-threshold powers in miRNA expression matrix； C, Analysis of the scale-free fit index for various soft-threshold powers in mRNA expression matrix; the red line was set at 0.80. D, Analysis of mean connectivity for various soft-threshold powers in mRNA expression matrix.

Figure S2. Functional KEGG enrichment analysis of HCM-related mRNA modules

Figure S3. Top 5 characteristic variables filtering based on RF

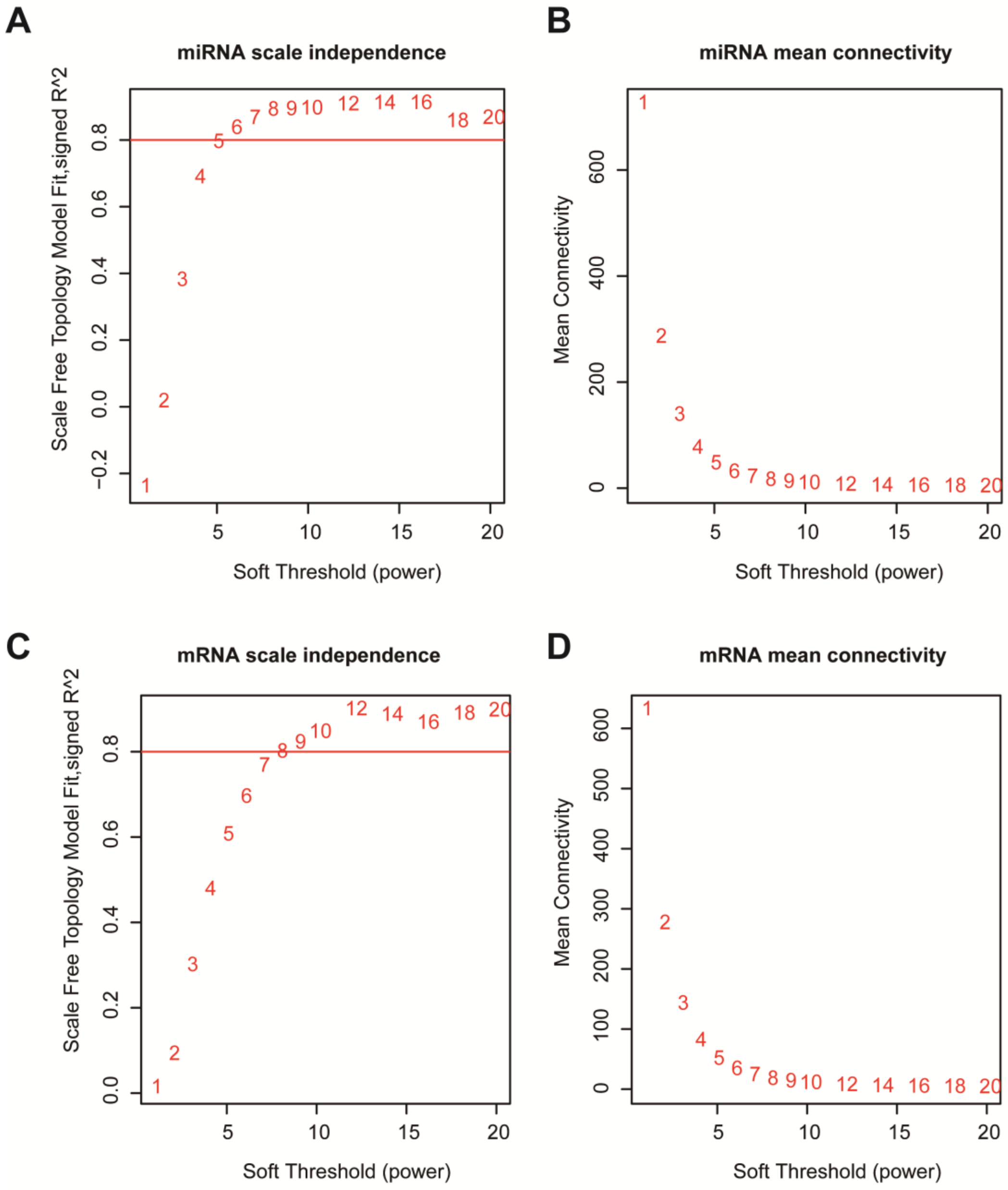

Figure S1. Effects of different power values on the scale independence degree and mean connectivity of co-expression modules

A, Analysis of the scale-free fit index for various soft-threshold powers in miRNA expression matrix; the red line was set at 0.80. B, Analysis of mean connectivity for various soft-threshold powers in miRNA expression matrix; C, Analysis of the scale-free fit index for various soft-threshold powers in mRNA expression matrix; the red line was set at 0.80. D, Analysis of mean connectivity for various soft-threshold powers in mRNA expression matrix.

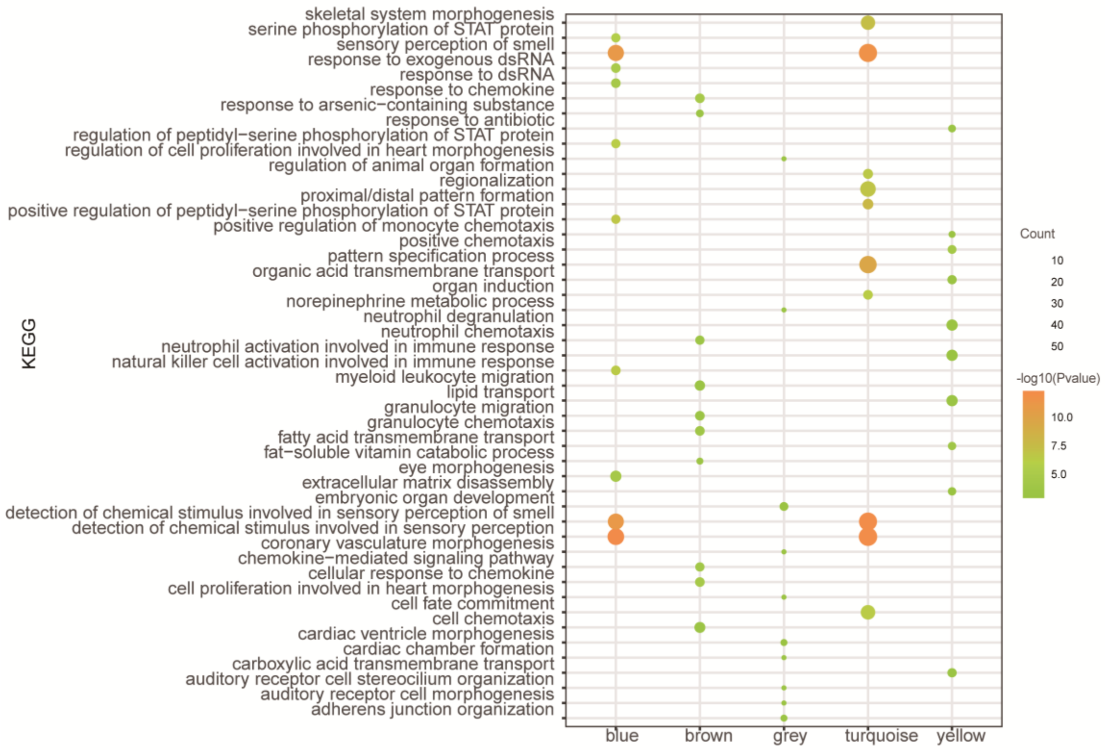

Figure S2. Functional KEGG enrichment analysis of HCM-related mRNA modules

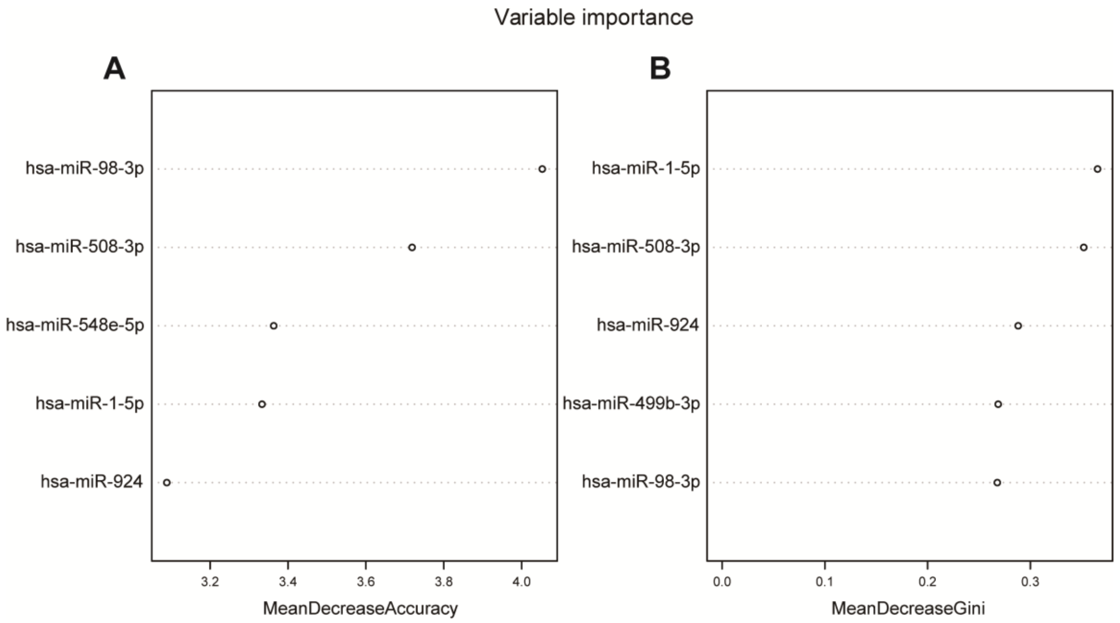

Figure S3. Top 5 characteristic variable filtering based on RF

A. Top 5 mean decrease accuracy miRNAs based on RF. B. Top 5 mean decrease Gini miRNAs based on RF.

Additional file tables

Table S1. Primer sequences for miRNAs

Gene name	5'-3' sequence
hsa- miR-508-3p	GCTGATTGTAGCCTTTTGGAGTAGA
hsa- miR-548e-5p	CAAAAGCAATCGCGGTTTTT
hsa- miR-1-5p	GGCACATACTTCTTTATATGCCCAT
hsa- miR-924	GGCAGAGTCTTGTGATGTCTTGC
hsa- miR-499b-3p	GCAACATCACTGCAAGTCTTAACA
hsa- miR-629-5p	TGGGTTTACGTTGGGAGAACT
hsa- miR-520f-3p	CAAGTGCTTCCTTTTAGAGGGTT
hsa- miR-450a-2-3p	ATTGGGGACATTTTGCATTCAT
hsa- miR-325	GCCCTAGTAGGTGTCCAGTAAGTGT
hsa- miR-98-3p	CGCGGCCTATACAACTTACTACTTTCCC
hsa- miR-3613-5p	CGGCTGTTGTACTTTTTTTTTTGTTC
hsa- miR-572	GTCCGCTCGGCGGTGGC

Table S2. DEGs in HCM compared with NC groups

Table S3. miRNAs in miRNA-Modules

Table S4. mRNAs in mRNA-Modules

（see supplementary Tables.xlsx）
